# Complete mitochondrial genome of marine Petrale sole *Eopsetta jordani* (Pleuronectiformes: Pleuronectidae) flatfish

**DOI:** 10.1080/23802359.2022.2080019

**Published:** 2022-06-10

**Authors:** Maheshkumar Prakash Patil, Jong-Oh Kim, Yong Bae Seo, Jiyoung Shin, Ji-Young Yang, Gun-Do Kim

**Affiliations:** a Industry-University Cooperation Foundation, Pukyong National University, Busan, Republic of Korea; b Department of Microbiology, Pukyong National University, Busan, Republic of Korea; c School of Marine and Fisheries Life Science, Pukyong National University, Busan, Republic of Korea; d Research Institute for Basic Science, Pukyong National University, Busan, Republic of Korea; e Institute of Food Science, Pukyong National University, Busan, Republic of Korea; f Department of Food Science and Technology, Pukyong National University, Busan, Republic of Korea

**Keywords:** *Eopsetta jordani*, Pleuronectidae, mitochondrion genome, phylogenetic analysis

## Abstract

Petrale sole *Eopsetta jordani* (Pleuronectiformes: Pleuronectidae) is a species of flounder, found in the northeastern Pacific Ocean and the Bering Sea of the United States and Canada. The complete mitochondrial DNA (mtDNA) of *E. jordani* has 16,483 bp with an overall A + T content of 61% and consists of 2 ribosomal RNA (rRNA) genes, 13 protein-coding genes (PCGs), 22 transfer RNA (tRNA) genes, and a non-coding control region. It has incomplete stop codon genes in *ND2*, *COII*, *ATPase6*, *COIII*, *ND3*, and *ND4*. Phylogenetic analysis indicated that *E. jordani* is not monophyletic with cogeneric *Eopsetta grigorjewi* and is separated from other species in the same family by a large distance. Present study results provide useful data for further research on genetic diversity and evolution of the *Eopsetta* and the Pleuronectidae.

The *Eopsetta jordani* (Lockington, 1879) is a bony edible fish commonly known as Petrale sole, belonging to the family Pleuronectidae. It is a righteye flounder with an oval body and light to dark brown upper surface. The genus *Eopsetta* contains two species, namely, *E. grigorjewi* (Herzenstein, 1890) and *E. jordani* (Cooper and Chapleau 1998). Petrale sole is found in the northeastern Pacific Ocean and along the coast of the Bering Sea from Alaska to Coronado Island, Baja California, Mexico, and Canada (Kramer et al. 1995; Love et al. 2005). It is an important commercial demersal flatfish (Love et al. 2005; Wetzel 2019). The female Petrale sole (63 cm on average) is bigger than the male (an average, 50 cm) (Pedersen 1975). Because commercially driven misnaming with similar morphological species is a growing concern in Korea, it is critical to developing improved bio-techniques to determine the quality or originality of fishes, which requires molecular-level study. As a result, the current work describes the analysis of *Eopsetta jordani* full mtDNA, with the goal that it will help with molecular assessment, construction of a biomarkers database, and research in the future.

The specimen was caught on the Busan coast in the Republic of Korea (35°07′10.8′′N, 129°10′32.7′′E). The voucher specimen was deposited to the Food Engineering Department in the Pukyong National University, Busan, Republic of Korea (Ji-Young Yang, jyyang@pknu.ac.kr) with specimen number MFDS-FSE01. DNeasy Blood and Tissue Kit (Qiagen, Germany) was used to extract genomic DNA from muscle following the manufacturer’s instructions. DNA library was constructed using MGIEasy DNA Library Prep Kit (MGI, China) and sequenced on MGISEQ-2000 Sequencing System (MGI) with paired-end reads (150 bp). The obtained sequence read-pairs were cleaned using Cutadapt ver. 1.9 (Martin 2011) before *de novo* assembly using CLC Genomics Workbench (Qiagen, Germany). The final full mtDNA sequence annotation was performed using the MitoFish pipeline (Sato et al. 2018). Reference sequences of the related Pleuronectidae species and outgroup species were downloaded from GenBank, and the phylogenetic analysis was conducted applying the Maximum Likelihood Method and Tamura-Nei model with 1000 bootstrap in MEGA11 (Tamura et al. 2021).

The complete and closed-circular mitochondrial genome (mtDNA) of *E. jordani* with a length of 16,483 bp was submitted to GenBank (https://www.ncbi.nlm.nih.gov) under the accession number OK545541.1. The mtDNA base composition contains A 32%, T 29%, G 14%, and C 25%, with G + C contents 39%. The mtDNA contains a total of 38 genes, including 2 rRNA, 13 protein-coding genes (PCGs), 22 tRNA, and a non-coding control region. Among all genes, *ND6* and 7 tRNA (*Pro*, *Glu*, *Ser*, *Tyr*, *Cys*, *Asn*, and *Ala*) genes were positioned on the L-strand. There are incomplete stop codon genes in ND2, COII, ATPase6 (*atp6*), COIII, ND3, ND4, and atypical codon usage in tRNA-Leu (UAA, UAG) and tRNA-Ser (UGA, GCU) were observed.

A phylogenetic tree was constructed using sequences from 13 published mtDNA of the family Pleuronectidae with *E. jordani* (Figure 1). The results show that *E. jordani* (OK545541.1) is not monophyletic with cogeneric *E. grigorjewi* (OK545542.1) and is separated from other species in the same family by a large distance. For a better understanding of the species’ phylogeny, a detailed morphological and molecular phylogenetic investigation is required. The present study findings provide useful data for further research on genetic diversity and the evolution of *Eopsetta* and Pleuronectidae.

**Figure 1.**
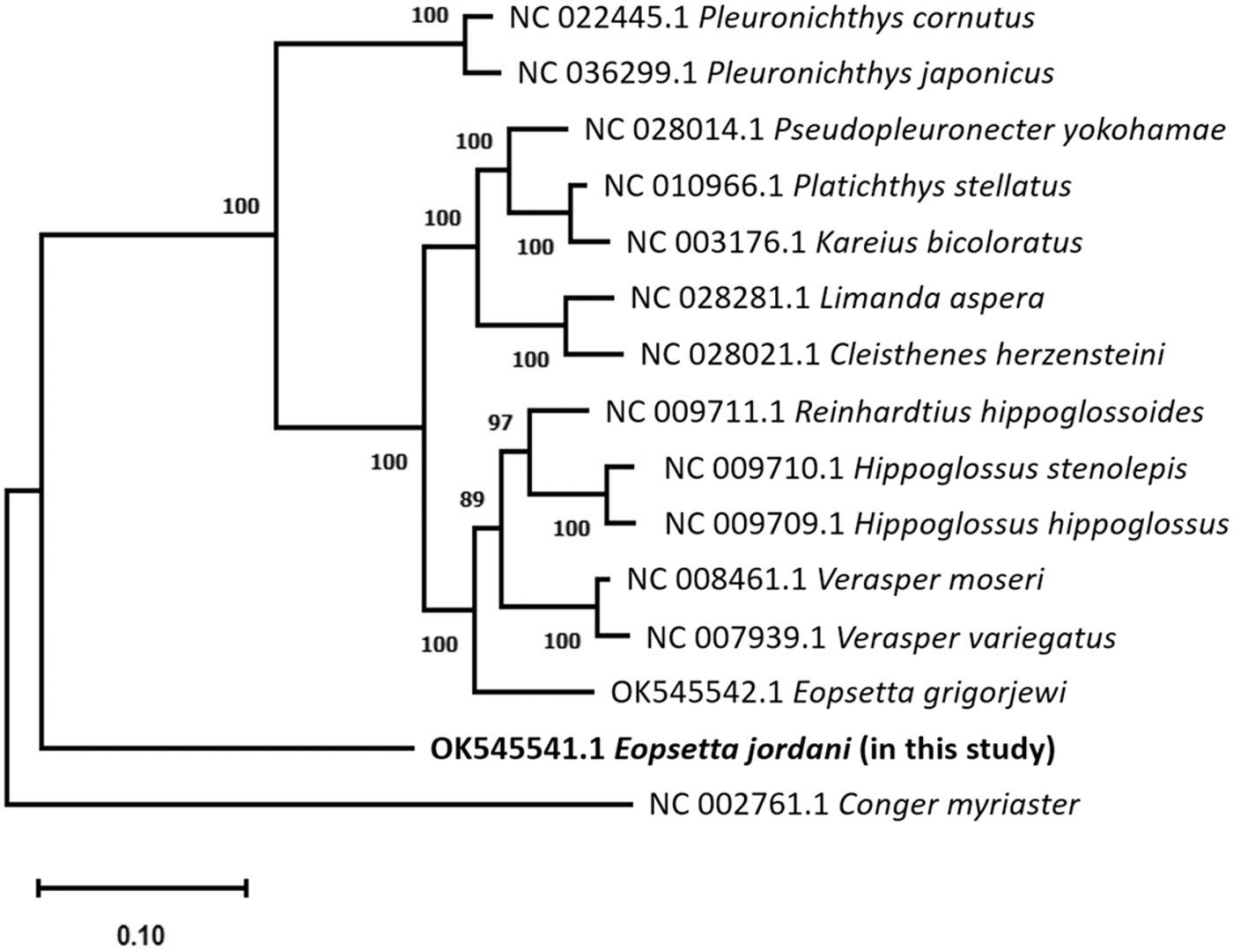
The phylogenetic tree shows a relationship between *Eopsetta jordani* and thirteen Pleuronectidae species, with *Conger myriaster* as the outgroup, based on mitogenome sequences from GenBank, sequences aligned by ClustalW using Maximum-likelihood method, and Tamaru–Nei model with 1000 bootstrap replicates. The numbers (%) in each node represent the bootstrap possibilities.

## Data Availability

The genome sequence data that support the findings of this study are openly available in GenBank of NCBI at (https://www.ncbi.nlm.nih.gov/) under accession no. OK545541. The associated BioProject, BioSample, and SRA numbers are PRJNA794310, SAMN24622875, and SRP353556, respectively.
